# Gender-specific relationship between frequency of food-away-from-home with serum lipid levels and dyslipidemia in chinese rural adults

**DOI:** 10.1186/s12944-022-01719-6

**Published:** 2022-11-01

**Authors:** Yuyang Wang, Xiaotian Liu, Xiaokang Dong, Beibei Liu, Ning Kang, Wenqian Huo, Zhenxing Mao, Jian Hou, Chongjian Wang

**Affiliations:** Department of Epidemiology and Biostatistics, College of Public Health, Zhengzhou University, 100 Kexue Avenue, 450001 Zhengzhou, Henan PR China

**Keywords:** Gender-specific, Food-away-from-home, Serum lipid levels, Dyslipidemia

## Abstract

**Objective:**

Food-away-from-home (FAFH) is one of the leading dietary patterns in Chinese families. However, the relationship between FAFH and dyslipidemia remains unclear, especially in the rural adult population. This study explored the relationship of FAFH frequency with serum lipid levels and dyslipidemia in rural Chinese adults.

**Methods:**

A total of 12,002 men and 17,477 women aged 18–79 were included from the Henan rural cohort. Serum lipid levels were measured by enzyme colorimetry. Information on FAFH frequency was collected using a validated questionnaire. The associations of FAFH frequency and serum lipid levels were assessed through multiple linear regression modeling. Logistic regression was performed to explore the linkages of the FAFH frequency to dyslipidemia and its four parameter types. Mediation analysis examined whether body mass index (BMI) acted as a mediator between the FAFH frequency and dyslipidemia.

**Results:**

After adjusting for potential confounders, the adjusted odds ratio (*OR*) and 95% confidence interval (*CI*) of the groups with 8–11 FAFH times/week for dyslipidemia were 1.991 (1.569, 2.526) in men compared with 0-frequency subgroup. Participants who consumed 8–11 FAFH times/week had a higher risk of high total cholesterol (TC), high triglycerides (TG), high LDL-cholesterol (LDL-C), and low HDL-cholesterol (HDL-C) with the *OR* and 95% *CI* of 1.928 (1.247, 2.980), 1.723 (1.321, 2.247), 1.875 (1.215, 2.893), and 1.513 (1.168, 1.959), respectively. In addition, the interaction effect between FAFH and gender was significantly associated with dyslipidemia and lipid levels (*P* < 0.001). BMI played a fully mediating effect between FAFH frequency and dyslipidemia in men, and the Sobel test showed the significance of the mediating effect (z = 4.2158, *P* < 0.001).

**Conclusion:**

In rural Chinese adults, FAFH was significantly associated with a higher risk of dyslipidemia, which indicated the importance of FAFH reduction and dietary intervention in patients with dyslipidemia and cardiovascular disease, especially in clinical practice.

**Trial Registration:**

The Henan Rural Cohort Study has been registered on the Chinese Clinical Trial Register (Registration number: ChiCTR-OOC-15,006,699).

**Supplementary Information:**

The online version contains supplementary material available at 10.1186/s12944-022-01719-6.

## What is already known on this topic?

Earlier studies have explored the relationship between food-away-from-home (FAFH) and metabolic diseases, including obesity, diabetes, etc. Still, little has focused on the association between the frequency of FAFH with serum lipid levels and dyslipidemia. A limitation of these studies was the small sample size and little focus on rural areas. FAFH is increasingly common in rural areas as China’s economy develops rapidly. However, the evidence for the association between the frequency of FAFH and dyslipidemia is still limited and needs further exploration.

## What does this study add?

The present study explored the relationship between FAFH and serum lipid levels and further analyzed the relationship between FAFH and dyslipidemia and its components. Results showed that FAFH was significantly associated with higher levels of serum total cholesterol (TC), triglycerides (TG), low-density lipoprotein cholesterol (LDL-C) and lower high-density lipoprotein cholesterol (HDL-C), as well as a higher risk of dyslipidemia. These associations were observed in men and rarely in women. Furthermore, this study identified whether body mass index (BMI) acted as a mediator between the FAFH frequency and the dyslipidemia.

## Background

Cardiovascular disease (CVD) carries a heavy disease burden worldwide, with age-standardized rates of CVD beginning to rise in almost all countries, including high-income countries [1]. CVD is the leading cause of death in urban and rural areas, accounting for 46.66% in rural areas and 43.81% in urban areas [2]. Due to China’s rapid economic development and lifestyle changes, the overall age-standardized prevalence of CVD has increased significantly by 14.7% from 1990 to 2016 [3]. As a significant risk factor for CVD, it was recently reported that the prevalence of dyslipidemia is 34.0% overall, 35.1%, and 26.3%, respectively, in rural and urban areas, while the treatment and control rates of dyslipidemia remained low, especially in rural China [4, 5]. Long-term prospective epidemiological studies have consistently shown that many modifiable factors influence dyslipidemia [6–8]. Studies found that preventing dyslipidemia and properly managing lipid levels could significantly alter cardiovascular mortality [9, 10]. A healthy diet, regular exercise, and avoiding smoking and alcohol can help reduce dyslipidemia and CVD risks [11–14].

With the rapid growth of China’s food and beverage industry, more and more residents’ dietary consumption mode has changed from self-supporting families to food-away-from-home (FAFH). It was reported that in 2012, 42.2% of urban residents and 28.5% of rural residents ate out, respectively. A systematic review indicated that FAFH might be linked to higher energy, fat intake, and lower micronutrient intake [15]. Serval research has found that FAFH was associated with unhealthy outcomes like obesity [16, 17] and type 2 diabetes mellitus (T2DM) [18], but evidence on the risk of dyslipidemia due to FAFH has been limited and mixed. A cross-sectional survey of 1,415 people showed that participants with hyperlipidemia were more likely to eat out regardless of gender [19]. As reported by Wang et al., FAFH decreased the risk of high triglycerides (TG) in young women and increased the risk for middle-aged men [20]. A small sample study in Korea found that consuming FAFH more frequently was associated with a lower risk of high serum total cholesterol (TC) and high LDL-cholesterol (LDL-C), but not low HDL-cholesterol (HDL-C) [21]. The limitations of these studies are their small sample size and lack of focus on rural FAFH-related dyslipidemia. The association between the frequency of FAFH and dyslipidemia remains unclear and needs further exploration.

Henan Province is a major agricultural production province with a population of about 94 million in 2010 (61.4% of rural areas), which is a typical representative of China’s rural areas [22]. In 2009, the per capita consumption of FAFH in Henan Province was 184.68 renminbi (RMB), accounting for 5.5% of the total food consumption expenditure [23]. In addition, some studies showed that the per capita intake of cereals and vegetables in Henan Province continued to decline, while the per capita intake of potatoes, sweet foods, and snacks showed a significant upward trend, which might be associated with the rising proportion of FAFH consumption [24]. Therefore, this study aimed to explore the associations of dyslipidemia and FAFH frequency, which presented important guidance for dietary care of patients with clinical dyslipidemia.

## Materials and methods

### Study population

From July 2015 to September 2017, the Henan Rural Cohort Study was carried out in 5 rural cities/counties of Henan, namely Tongxu, Yima, Suiping, Xinxiang, and Yuzhou, separately represent the provincial eastern, western, southern, northern, and central areas [25]. Backed up by the Centers for Disease Control and Prevention and the regional health sector, 18–79 years-old people were enrolled into the present cohort work through the multi-stage stratified probability sampling. In total, the baseline survey was filled out by 39,259 subjects, and the corresponding recovery was 93.7%. Since the dietary nutrition survey was not conducted in Yuzhou in 2015, subjects in Yuzhou (*n* = 9,237) and those with missing data on FAFH frequency (*n* = 37) and blood lipid levels (*n* = 197) in other four counties, as well as those suffering from cancer (*n* = 291) or serious renal condition (*n* = 18) were excluded. Ultimately, 12,002 men and 17,477 women were included into the analysis, totaling 29,479 subjects. The Henan Rural Cohort, which conformed to the guidelines of the Helsinki Declaration, was approved by the Life and Science Ethics Committee of Zhengzhou University. In addition, an informed consent in written form was acquired from every subject. Details of the research design, protocols and the traits of subjects can be found in the former work [25].

### Laboratory measurement

Following a minimum of 8-h overnight fasting, the acquisition of fasting venous blood was accomplished in the absence of anticoagulation for every subject using a vacuum tube. For isolation of serum samples from the whole blood, a 10-min centrifugation (3,000 rpm) was performed at ambient temperature. The Cobas c501 enzymatic analyzer (Roche, Switzerland) was utilized for the determination of lipid levels (including TC, TG, HDL-C, and LDL-C) by enzyme colorimetry.

### Definition of FAFH and dyslipidemia

FAFH was referred to herein as any food acquired from food plazas, fast food outlets, takeaway restaurants, food stalls, or groceries. In the course of the baseline survey, the subjects answered FAFH-related questions from the interviewers, including “How many times on average did you eat out for three meals weekly?” The weekly frequencies of FAFH were classified into 0, 1–3, 4–7, 8–11, and ≥ 12 meals [18, 26].

The 2016 Modified Guidelines for the Dyslipidemia Prevention and Treatment among Chinese Population stipulate that the thresholds for high TC and TG were 6.22 mmol/L (240 mg/dL) and 2.26 mmol/L (200 mg/dL), respectively, while the thresholds for high LDL-C and low HDL-C were separately 4.14 mmol/L (160 mg/dL) and 1.04 mmol/L (40 mg/dL). Any subject was regarded as dyslipidemic when single or multiple abnormalities in blood levels of lipids were present, or antidyslipidemics were taken in the past 14 days [27].

### Assessment of covariates

For the acquisition of specific data concerning the demographic information (age, gender, marital status, educational level, and mean monthly income), lifestyle choices (alcohol drinking, smoking, physical activity, history of diseases), as well as dietary habits (high-fat diet, consumption of fruits and vegetables). Face-to-face interviews were conducted, where the subjects answered regular survey questions from the well-trained assistant researchers. Alcohol drinking herein referred to a minimum of 12 drinks on a yearly basis, whereas smoking referred to a minimum of 1 cigarette daily for 6 consecutive months. Physical activity was categorized as high, moderate or low by the International Physical Activity Questionnaire (IPAQ) [28]. Adequate fruit and vegetable intake herein referred to the daily consumption of over 500 g per person. Any person was considered to have high-fat diet when his/her daily intake of livestock and poultry meat exceeds 75 g [29]. A familial dyslipidemia history was indicated as a history of dyslipidemia in the subjects’ parents or siblings. In addition, the computational formula for body mass index (BMI) was the weight of the body (kg) divided by the square of height (m). The height, weight and waist circumference (WC) of each subject was measured twice by the well-trained research staff.

### Statistical analysis

Student’s t-test was used to compare continuous variables that satisfy normality and were expressed as means ± standard deviations (SDs), non-normal distributed continuous variables were presented as median with interquartile ranges (IQRs) and analyzed with Mann-Whitney U test. Meanwhile, χ² test was introduced for comparison of categorical variables, which were expressed as percentages and numbers. To satisfy the normal distribution of lipid levels (TC, TG, HDL-C and LDL-C), multiple linear regression was used to evaluate the relationship between FAFH frequency and naturally log-transformed lipid levels, and the percentage changes with the corresponding 95% *CIs* were correlated through the back-transforming effect using 100 × [exp (β) − 1] [30]. Using the 0 FAFH frequency group as the reference group, Logistic regression was performed to assess the linkages of the FAFH frequency to the dyslipidemia and its 4 parameter types by odds ratios (*ORs*) and 95% *CIs*. Among the 3 models subjected to analysis in the current work, the Model 1 was adjusted for age and gender (only for total participants), the Model 2 was adjusted for age, gender (only for total participants), educational level, marital status, mean monthly income, alcohol consumption and smoking, and the Model 3 was adjusted further for consumption of fruits and vegetables, high-fat diet, physical activity and familial dyslipidemia history. FAFH-gender was used as an interaction term to explore the effect of the interaction between FAFH and gender on dyslipidemia and blood lipid levels. To examine whether BMI acted as a mediator between the FAFH frequency and the dyslipidemia among men subjects, and the Sobel test was used to assess the significance of the mediating effect [31]. Mediation analysis was performed with the aid of PROCESS in SPSS. The computational formula for the proportional effect of BMI in the mediation analysis was: (*β*_indirect effect_/ *β*_total effect_) × 100%. Besides, the FAFH-dyslipidemia correlation in various subgroups was explored by the stratified analysis. With the aid of SPSS 21.0 software and R version 3.6.3, the *P* values were all two-tailed at the 0.05 level of significance.

## Results

### Basic traits of the population

**Fig. 1:**
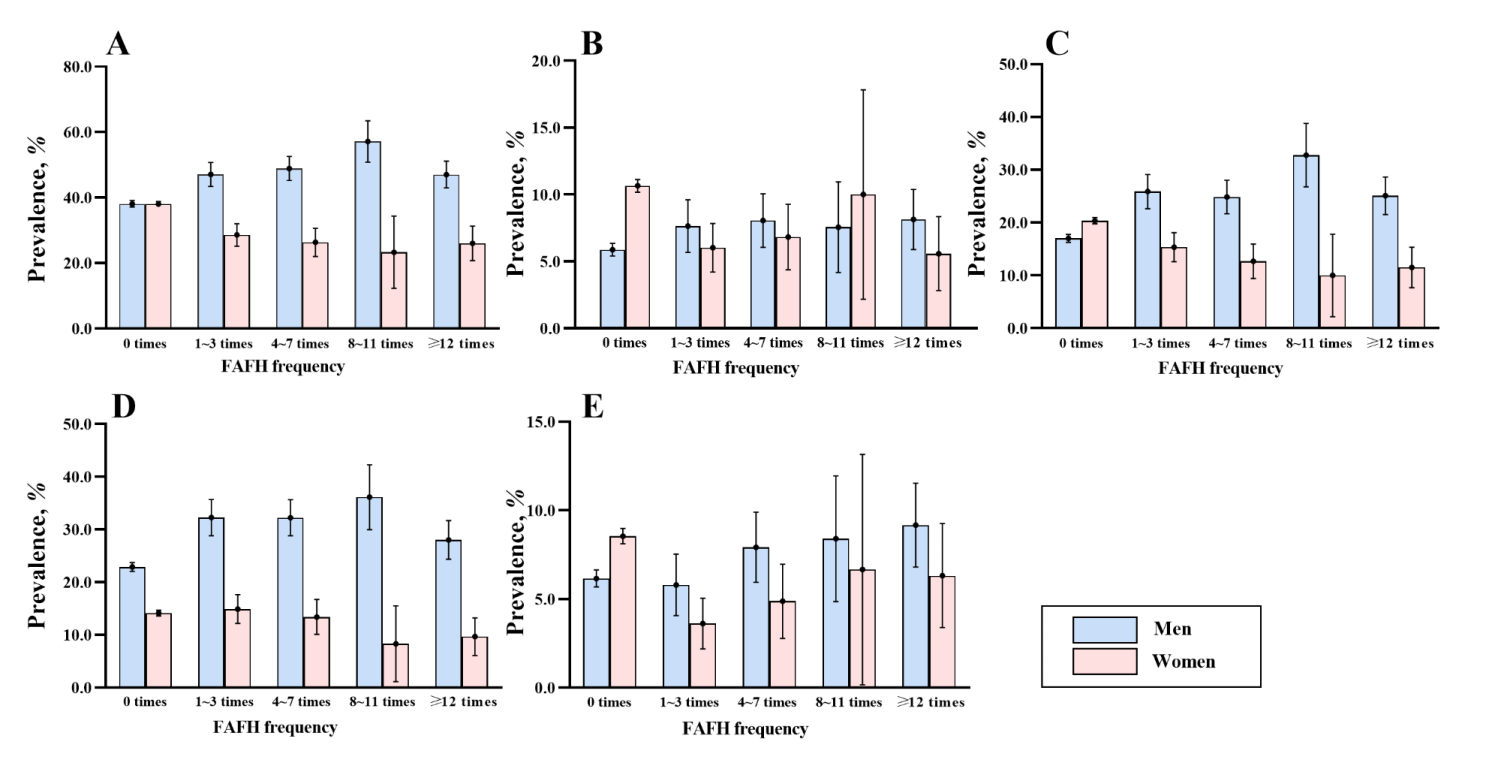
Crude prevalence of dyslipidemia (**A**), high TC (**B**), high TG (**C**), low HDL-C (**D**), and high LDL-C (**E**) in different FAFH frequency group by gender

According to the basic trait information for the subjects detailed in Table 1, 11,312 of 29,479 subjects had dyslipidemia, with a crude prevalence of 38.4% (40.1% among men and 25.7% among women). Lower mean monthly income and levels of education, consumption of vegetables and fruits and physical activity, alcohol takers and smokers were more likely to have dyslipidemia (*P* < 0.05). Overall, the mean FAFH frequency was 0.9 ± 3.2 on a weekly basis. The weekly frequency of FAFH in the general population was 0.9 ± 3.2, 1.5 ± 4.0 in men, and 0.5 ± 2.4 in women. The frequency of FAFH in men was significantly higher than in women (*P* < 0.001). Figure 1 depicts the gender-wise data of crude dyslipidemia prevalence and four parameter types.

**Table 1:** Characteristics of participants according to dyslipidemia

Characteristics	Total (n = 29,479)			Men (*n* = 12,002)			Women (*n* = 17,477)		
	**Dyslipidemia**	**No − dyslipidemia**	***P***	**Dyslipidemia**	**No − dyslipidemia**	***P***	**Dyslipidemia**	**No − dyslipidemia**	***P***
**Age**, ***n*(**%**)**	56.4 ± 11.5	54.8 ± 12.8	< 0.001	54.8 ± 12.4	57.6 ± 12.3	< 0.001	57.5 ± 10.7	53.0 ± 12.9	< 0.001
**Marital status**, ***n*(**%**)**			0.381			< 0.001			0.002
Married/cohabitation	10,216 (90.3)	16,350 (90.0)		4420 (91.9)	6404 (89.1)		5796 (89.2)	9946 (90.6)	
Unmarried/divorced/widowed	1096 (9.7)	1817 (10.0)		392 (8.1)	786 (10.9)		704 (10.8)	1031 (9.4)	
**Education level**, ***n*(**%**)**			< 0.001			< 0.001			< 0.001
Elementary school or below	9472 (83.8)	15,051 (82.8)		3650 (75.9)	5780 (80. 3)		5822 (89.5)	9271 (84.4)	
Junior high school	1521 (13.4)	2429 (13.4)		939 (19.5)	1183 (16.5)		582 (9.0)	1246 (11.4)	
High school or above	319 (2.8)	687 (3.8)		223 (4.6)	227 (3.2)		96 (1.5)	460 (4.2)	
**Averaged monthly income**, ***n*(**%**)**			0.191			< 0.001			< 0.001
< 500RMB	4087 (36.1)	6546 (36.0)		1616 (33.6)	2777 (38.6)		2471 (38.1)	3769 (34.3)	
500–999 RMB	3622 (32.0)	5668 (31.2)		1500 (31.2)	2170 (30.2)		2122 (32.6)	3498 (31.9)	
≥ 1000RMB	3603 (31.9)	5953 (32.8)		1696 (35.2)	2243 (31.2)		1907 (29.3)	3710 (33.8)	
**Smoking**, ***n*(**%**)**			< 0.001			0.176			0.118
Never	7956 (70.3)	13,274 (73.0)		1489 (30.9)	2332 (32.4)		6467 (99.4)	10,942 (99.7)	
Ever	957 (8.5)	1357 (7.5)		947 (19.7)	1349 (18.8)		10 (0.2)	8 (0.1)	
Current	2399 (21.2)	3536 (19.5)		2376 (49.4)	3509 (48.8)		23 (0.4)	27 (0.2)	
**Drinking**, ***n*(**%**)**			< 0.001			0.014			0.150
Never	8629 (76.3)	14,276 (78.6)		2252 (46.8)	3554 (49.5)		6377 (98.1)	10,722 (97.7)	
Ever	608 (5.4)	841 (4.6)		594 (12.3)	816 (11.3)		14 (0.2)	25 (0.2)	
Current	2075 (18.3)	3050 (16.8)		1966 (40.9)	2820 (39.2)		109 (1.7)	230 (2.1)	
**Physical activity**, ***n*(**%**)**			< 0.001			< 0.001			< 0.001
Low	4043 (35.8)	5328 (29.3)		1917 (39.8)	2224 (30.9)		2126 (32.7)	10,722 (97.7)	
Moderate	4144 (36.6)	6700 (36.9)		1384 (28.8)	1994 (27.7)		2760 (42.5)	25 (0.2)	
High	3125 (27.6)	6139 (33.8)		1511 (31.4)	2972 (41.4)		1614 (24.8)	230 (2.1)	
**Vegetable and fruit intake**, ***n*(**%**)**			0.070			0.006			0.937
< 500 g/d	5976 (52.8)	9401 (51.7)		2579 (53.6)	3671 (51.1)		3397 (52.3)	5730 (52.2)	
≥ 500 g/d	5335 (47.2)	8766 (48.3)		2232 (46.4)	3519 (48.9)		3103 (47.7)	5247 (47.8)	
**High fat diet**, ***n*(**%**)**	2017 (17.8)	3330 (18.3)	0.279	1205 (25.0)	1634 (22.7)	0.003	812 (12.5)	1696 (15.5)	< 0.001
**Family history of dyslipidemia**, ***n*(**%**)**	473 (4.2)	603 (3.3)	< 0.001	173 (3.5)	182 (2.5)	0.005	309 (4.8)	421 (3.8)	0.003
**BMI (mean ± SD)**	25.9 ± 3.4	24.0 ± 3.5	< 0.001	25.9 ± 3.2	25.9 ± 3.2	< 0.001	25.9 ± 3.5	24.3 ± 3.6	< 0.001
**WC (mean ± SD)**	87.6 ± 9.8	81.2 ± 10.1	< 0.001	89.8 ± 9.7	82.3 ± 10.0	< 0.001	85.9 ± 9.6	80.5 ± 10.1	< 0.001
**Frequency of FAFH (times/week)**	1.0 ± 3.3	0.9 ± 3.2	0.012	1.8 ± 4.3	1.8 ± 3.8	< 0.001	0.4 ± 2.1	0.6 ± 2.6	< 0.001
**TC (mmol/L)**	5.1 (4.3, 6.1)	4.6 (4.1, 5.2)	< 0.001	4.9 (4.1, 5.7)	4.5 (4.0, 5.1)	< 0.001	5.4 (4.5, 6.3)	4.7 (4.2, 5.2)	< 0.001
**TG (mmol/L)**	2.3 (1.5, 3.0)	1.2 (0.9, 1.5)	< 0.001	2.1 (1.4 2.9)	1.1 (0.9, 1.5)	< 0.001	2.3 (1.6, 3.0)	1.2 (0.9, 1.5)	< 0.001
**HDL − C (mmol/L)**	1.1 (0.9, 1.3)	1.4 (1.2, 1.6)	< 0.001	1.0 (0.9, 1.2)	1.4 (1.2, 1.6)	< 0.001	1.2 (1.0, 1.4)	1.4 (1.3, 1.7)	< 0.001
**LDL − C (mmol/L)**	3.0 (2.4, 3.9)	2.8 (2.3, 3.2)	< 0.001	2.9 (2.3, 3.7)	2.7 (2.3, 3.2)	< 0.001	3.1 (2.4, 4.0)	2.8 (2.3, 3.2)	< 0.001

Continuous variables with normal distribution are presented as mean ± SD and analyzed with t-test, non-normal distributed continuous variables are presented as median (IQR) and analyzed with Mann-Whitney U test; categorical variables are shown as numbers (%) and analyzed with chi-square test

***BMI*** body mass index; ***WC*** waist circumference; ***RMB*** renminbi; ***FAFH*** food-away-from-home; ***TC*** total cholesterol; ***TG*** triglycerides; ***HDL − C*** high − density lipoprotein cholesterol; ***LDL − C*** low − density lipoprotein cholesterol; ***SD*** standard deviation; ***IQR*** interquartile range

### Association of FAFH frequency with serum lipid levels

Table 2 shows the association of FAFH frequency with serum lipid levels. Higher FAFH frequencies were significantly associated with higher TC, TG, LDL-C and lower HDL-C levels in total and in men. When FAFH frequency is 8–11 times/week, the increment of FAFH frequency every 1 time/week was related to 7.144% (95% *CI*: 4.812%, 9.636%) increase in TC, 17.234% (95% *CI*: 10.296%, 24.608%) increase in TG, 4.603% (95% *CI*: 1.106%, 8.220%) increase in LDL-C, -4.305% (95% *CI*: -7.040%, -1.489%) decrease in HDL-C, respectively. In addition, a significant interaction between FAFH and gender was observed in the serum lipid levels (*P* < 0.001).

**Table 2:** Multivariable linear regression analysis of weekly frequency of food-away-from-home associated with serum lipid levels(mmol/L)

Weekly frequency of FAFH	TC			TG			HDL-C			LDL-C		
	% Changes	95% *CI*	*P*	% Changes	95% *CI*	*P*	% Changes	95% *CI*	*P*	% Changes	95% *CI*	*P*
**Total (*n*** **= 29,479)**												
0 time (*n* = 25,833)	0 (Ref.)			0 (Ref.)			0 (Ref.)			0 (Ref.)		
1 ~ 3 times (*n* = 1,371)	1.816	(0.702, 2.942)	0.001	1.816	(-0.110, 4.917)	0.216	-2.469	(-3.825, -1.193)	< 0.001	0.200	(-1.489, 1.816)	0.826
4 ~ 7 times (*n* = 1,130)	4.081	(2.840, 5.338)	< 0.001	4.289	(1.005, 7.681)	0.011	-3.149	-4.591, -1.686)	< 0.001	3.769	(1.918, 5.654)	< 0.001
8 ~ 11 times (*n* = 298)	7.144	(4.812, 9.636)	< 0.001	17.234	(10.296, 24.608)	< 0.001	-4.305	(-7.040, -1.489)	0.003	4.603	(1.106, 8.220)	0.010
≥ 12 times (*n* = 847)	2.737	(1.410, 4.185)	< 0.001	1.005	(-2.664, 4.707)	0.609	-0.896	(-2.566, 0.904)	0.322	3.977	(1.816, 6.184)	< 0.001
**Men (*n*** **= 12,002)**												
0 time (*n* = 9759)	0 (Ref.)			0 (Ref.)			0 (Ref.)			0 (Ref.)		
1 ~ 3 times (*n* = 707)	1.816	(0.200, 3.355)	0.024	6.078	(1.816, 10.517)	0.691	-3.439	(-5.351, -1.587)	< 0.001	0.401	(-1.882, 2.737)	0.719
4 ~ 7 times (*n* = 720)	3.355	(1.816, 5.022)	< 0.001	4.394	(0.200, 8.763)	0.002	-3.052	(-4.877, -1.094)	0.002	2.532	(0.200, 4.917)	0.035
8 ~ 11 times (*n* = 238)	4.603	(1.918, 7.358)	0.001	11.293	(3.873, 19.244)	0.041	-4.113	(-7.133, -0.896)	0.012	0.904	(-2.858, 4.917)	0.644
≥ 12 times (*n* = 578)	1.410	(-0.300, 3.149)	0.097	-0.896	(-5.351, 3.666)	0.005	-0.995	(-3.052, 1.207)	0.378	1.613	(-0.896, 4.289	0.207
**Women (*n*** **= 17,477)**												
0 time (*n* = 16,074)	0 (Ref.)			0 (Ref.)			0 (Ref.)			0 (Ref.)		
1 ~ 3 times (*n* = 664)	1.613	(0.000, 3.149)	0.044	-2.664	(-6.480, 1.410)	0.200	-1.587	(-3.536, 0.300)	0.100	-0.300	(-2.566, 2.122)	0.827
4 ~ 7 times (*n* = 410)	2.532	(0.602, 4.498)	0.011	-4.113	(-8.881, 0.904)	0.105	-1.784	(-4.209, 0.602)	0.138	2.429	(-0.499, 5.443)	0.100
8 ~ 11 times (*n* = 60)	2.840	(-2.078, 8.004)	0.264	-5.446	(-16.890, 7.681)	0.398	2.429	(-3.632, 8.981)	0.439	1.715	(-5.541, 9.527)	0.661
≥ 12 times (*n* = 269)	0.401	(-1.980, 2.737)	0.761	-9.968	(-15.380, -4.209)	0.001	2.224	(-0.698, 5.338)	0.243	2.429	(-1.094, 6.078)	0.182

***CI*** confidence interval;

Adjusted model for age, gender (only for total participants), marital status, education level, average monthly income, smoking status, drinking status, physical activity, vegetable and fruit intake, high fat diet, family history of dyslipidemia

### Associations of FAFH frequency with dyslipidemia and its components

Table 3 describes the weekly FAFH frequency-dyslipidemia correlation from the logistic regression outcomes. Following adjustment of potential confounders, the total and men groups exhibited statistically significant FAFH frequency-dyslipidemia incidence correlation (*P* < 0.05), but not for the women group (*P* > 0.05). In the total and men groups, progressive increases in the *OR* for dyslipidemia were noted with the heightening FAFH frequency (*P*_trend_ < 0.001). Besides, a significant interactive effect of gender and FAFH on dyslipidemia was observed in all three models (*P*_FAFH−gender_ < 0.001).

**Table 3:** Multivariate − adjusted *OR* and 95% *CI* for dyslipidemia according to weekly frequency of food-away-from-home

Weekly frequency of FAFH	*OR* (95% *CI*)			
	**Model 1**	**Model 2**	**Model 3**	*Per level risk
**Total (*n*** **= 29,479)**				1.016 (1.008, 1.024)
0 time (*n* = 25,833)	1 (Ref.)	1 (Ref.)	1 (Ref.)	
1 ~ 3 times (*n* = 1,371)	1.156 (1.030, 1.298)	1.133 (1.008, 1.273)	1.128 (1.004, 1.267)	
4 ~ 7 times (*n* = 1,130)	1.306 (1.151, 1.482)	1.295 (1.140, 1.471)	1.295 (1.139, 1.471)	
8 ~ 11 times (*n* = 298)	2.029 (1.604, 2.566)	1.983 (1.564, 2.514)	1.991 (1.569, 2.526)	
≥ 12 times (*n* = 847)	1.245 (1.078, 1.437)	1.241 (1.073, 1.434)	1.243 (1.075, 1.438)	
**Men (*n*** **= 12,002)**				1.014 (1.004, 1.024)
0 time (*n* = 9,759)	1 (Ref.)	1 (Ref.)	1 (Ref.)	
1 ~ 3 times (*n* = 707)	1.232 (1.053, 1.441)	1.219 (1.040, 1.427)	1.210 (1.032, 1.419)	
4 ~ 7 times (*n* = 720)	1.277 (1.091, 1.494)	1.265 (1.079, 1.483)	1.271 (1.083, 1.492)	
8 ~ 11 times (*n* = 238)	1.708 (1.309, 2.228)	1.703 (1.303, 2.226)	1.686 (1.287, 2.210)	
≥ 12 times (*n* = 578)	1.194 (1.004, 1.420)	1.177 (0.988, 1.401)	1.179 (0.988, 1.408)	
**Women (*n*** **= 17,477)**				0.985 (0.971, 1.000)
0 time (*n* = 16,074)	1 (Ref.)	1 (Ref.)	1 (Ref.)	
1 ~ 3 times (*n* = 664)	1.030 (0.860, 1.233)	1.050 (0.876, 1.259)	1.027 (0.856, 1.232)	
4 ~ 7 times (*n* = 410)	0.974 (0.773, 1.227)	0.997 (0.790,1.258)	0.996 (0.789, 1.257)	
8 ~ 11 times (*n* = 60)	0.948 (0.511, 1.757)	0.965 (0.519, 1.792)	0.979(0.525, 1.826)	
≥ 12 times (*n* = 269)	0.791 (0.596, 1.049)	0.806 (0.607, 1.070)	0.809 (0.609, 1.076)	
*P*_FAFH−gender_	< 0.001	< 0.001	< 0.001	

***CI*** confidence interval; ***OR*** odds ratio

Model 1: adjusted for age, gender (only for total participants)

Model 2: adjusted for age, gender (only for total participants), marital status, education level, average monthly income, smoking status, drinking status

Model 3: adjusted for age, gender (only for total participants), marital status, education level, average monthly income, smoking status, drinking status, physical activity, vegetable and fruit intake, high fat diet, family history of dyslipidemia

* Full − adjusted model for age, gender (only for total participants), marital status, education level, average monthly income, smoking status, drinking status, physical activity, vegetable and fruit intake, high fat diet, family history of dyslipidemia

***P***_FAFH−gender_ was performed using the interaction term, FAFH-gender, as a continuous variable in the logistic regression model

**Fig. 2:**
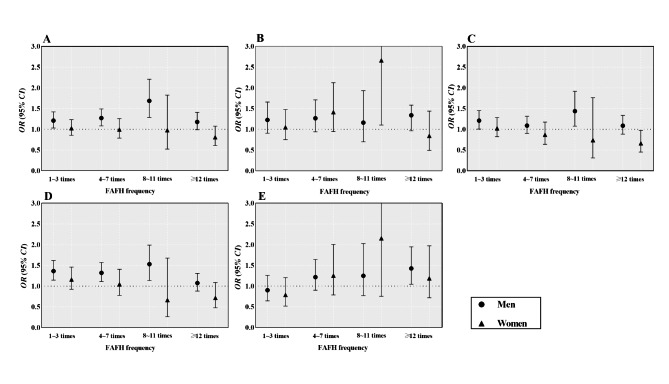
Association between FAFH frequency with dyslipidemia (**A**), high TC (**B**), high TG (**C**), low HDL-C (**D**), and high LDL-C (**E**) by gender. Abbreviations: *CI* confidence interval; *OR* odds ratio

The association of weekly FAFH frequency with the four types of abnormal serum levels of lipids was depicted in Fig. 2. In contrast to the 0 frequency subgroup, the self-reported weekly frequency of 8–11 FAFH meals was linked to higher probabilities of low HDL-C (*OR*: 1.503, 95% *CI*:1.136, 1.990) and high TC (*OR*: 1.438, 95% *CI*: 1.077, 1.920) among men. Besides, men with ≥ 12 frequency of FAFH were at higher risk of having high LDL-C (*OR*: 1.426, 95% *CI*: 1.044, 1.947). Additionally, compared with the 0-frequency subgroup, a higher probability of high TC was noted among women in the 8-11frequency subgroup (*OR*: 2.665, 95% *CI*: 1.105, 6.431). Table S1 lists the specific statistics of *OR* values and 95% *CIs*.

Table S6 reports the stratified analysis outcomes for various groups. Except for the men subjects, the FAFH consumption-dyslipidemia correlation was stronger among those aged ≥ 60 years than others in the present work *(OR*: 1.025, 95% *CI*: 1.006, 1.045). In contrast to the reference group, the dyslipidemia probability was also higher among the past and current alcohol takers and current smokers. Regarding physical activity, the dyslipidemia probability was kept higher among subjects with low and moderate levels of activity (*OR*: 1.022, 95% *CI*: 1.008, 1.036; *OR*: 1.017, 95% *CI*: 1.005, 1.030). Besides, the FAFH-dyslipidemia association was influenced significantly by the ≥ 500 g/d consumption of vegetables and fruits and a high-fat diet (*P* < 0.05).

### Association of the three-meal FAFH frequencies with dyslipidemia

**Fig. 3:**
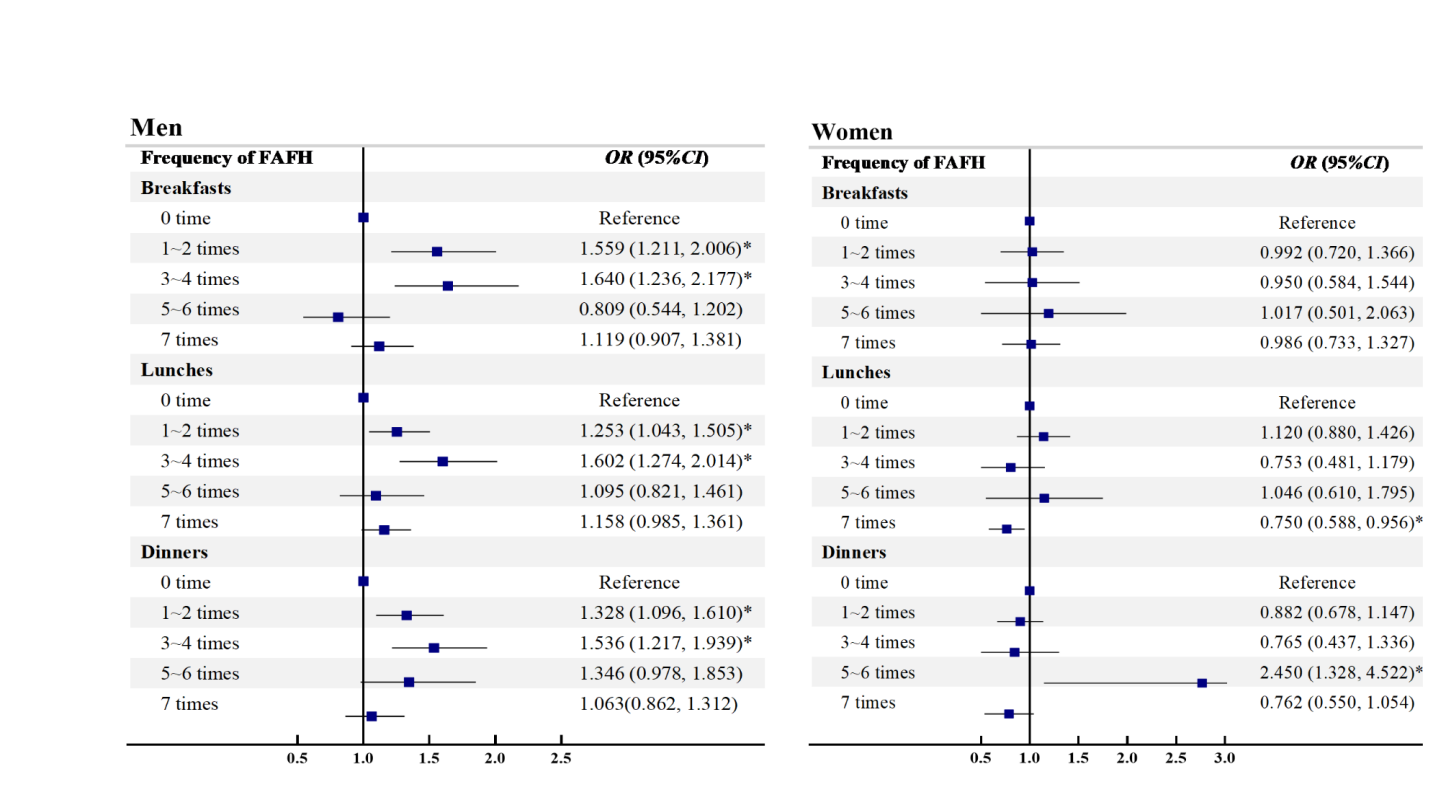
Association between frequency of eating out breakfasts, lunches and dinners with dyslipidemia by gender. ^*^P value < 0.05. Abbreviations: *CI* confidence interval; *OR* odds ratio. Full adjusted model for age, marital status, education level, average monthly income, smoking status, drinking status, physical activity, vegetable and fruit intake, high fat diet, family history of dyslipidemia

How the FAFH frequencies for three meals (breakfast, lunch and dinner) showed relationship to the dyslipidemia are described in Tables S2-S4 and Fig. 3. In contrast to the 0-frequency subgroup, eating out three meals was correlated significantly with a higher risk of dyslipidemia among the total population and men, especially for the 1–2 and 3–4 frequency subgroups (*P* < 0.05). Women who ate out dinner 5–6 times weekly were at higher dyslipidemic risk than those who did not eat out dinner (*OR*: 2.450, 95% *CI*: 1.328, 4.522). Besides, progressive increases in the *ORs* for dyslipidemia were noted with the heightening three-meal FAFH frequencies in the total and men (*P*_trend_ < 0.05).

### Mediation effects

The mediator role exerted by BMI between the FAFH frequency and the dyslipidemia is described in Table 4 for the men subjects. Following adjustment of potential confounders, the frequency of FAFH produced a significant gross impact on the dyslipidemia (*OR*: 1.014, 95% *CI*: 1.004–1.024). The association of higher BMI value with the FAFH frequency was significant, according to Table S5. Upon adoption of BMI as a mediator parameter, the FAFH exerted an insignificant direct impact on the dyslipidemia (*OR*: 1.007, 95% *CI*: 0.997–1.018), while the BMI-mediated indirect impact was found significant (*OR*: 1.008, 95% *CI*: 1.004–1.011). The Sobel test was significant (z = 4.2158, *P* < 0.001). Among the men subjects, BMI served as a complete mediator between the frequency of FAFH and the dyslipidemia. Additionally, a diagram of the mediation model is shown in Supplementary Fig. 1.

**Table 4:** Mediation analysis of the relationship between food-away-from-home frequency and dyslipidemia by BMI in men^a^

Mediation analysis	Parameter estimate (95% *CI*)	*OR* (95% *CI*)	*P*
Total effect	0.0139 (0.0040, 0.0237)	1.014 (1.004, 1.024)	0.0043
Direct effect path c′	0.0073 (-0.0029, 0.0175)	1.007 (0.997, 1.018)	0.1593
Path a	0.0343 (0.0185, 0.0501)	1.035 (1.019, 1.051)	< 0.001
Path b	0.2207 (0.2086, 0.2343)	1.247 (1.232, 1.264)	< 0.001
Indirect effect path ab	0.0076 (0.0040, 0.0112)	1.008 (1.004, 1.011)	0.0041

^a^ Adjusted for age, marital status, education level, average monthly income, smoking status, drinking status, physical activity, vegetable and fruit intake, high fat diet, family history of dyslipidemia

Path a indicates the path from the frequency of FAFH to BMI (Mediator)

Path b indicates the path from BMI to dyslipidemia

Path ab indicates the indirect effect of FAFH frequency on dyslipidemia mediated by BMI

Path c′ indicates the direct effect of FAFH frequency on dyslipidemia

***BMI*** body mass index, ***CI*** confidence interval, ***OR*** odds ratio

## Discussion

This study reported the relationship between the frequency of FAFH with serum lipid levels and dyslipidemia in rural China using a large sample of data, which provided scientific evidence for dietary intervention in dyslipidemia patients. This cross-sectional study found a significant association between the frequency of FAFH with serum lipid levels and dyslipidemia. Men, older, current smokers, past and current alcohol drinkers, and those who maintained low or moderate physical activity and high-fat diets were more at risk for dyslipidemia from FAFH. Moreover, BMI fully mediated between FAFH frequency and dyslipidemia in men.

This study found that FAFH frequency was significantly associated with higher TC, TG, LDL-C, and lower HDL-C levels and with a higher risk of dyslipidemia, which was mainly observed in men. Results of a study from the China Health and Nutrition Survey (CHNS) showed that middle-aged men with more frequent FAFH were associated with higher levels of serum TGs and lower HDL-C (*P* = 0.005), while a reverse association was observed in young women [20]. A survey in Yunnan-Guizhou Plateau in Southwest China also showed that a higher frequency of FAFH had a higher risk of hyperlipidemia [19]. Several factors contributed to the contradictory results between men and women caused by FAFH. Firstly, due to the traditional Chinese family living habits, women are mainly responsible for home cooking and food for the whole family. At the same time, men are more exposed to FAFH because of socializing at work or not being able to cook [32]. Besides, women pay more attention to health and wellness than men regarding dietary nutrition and lifestyle. In addition to the Chinese population, the lipid risk associated with FAFH has also been observed in other populations. A survey of Korean adults aged 20 and older found that those with FAFH frequency ≥ 7 times/week had a 32% higher likelihood of low HDL-C levels, compared to those with FAFH frequency < 1 time/week [33]. In addition, a study conducted among university students in Mexican found that students who had 1–2 times/weeks FAFH frequency were significantly associated with a four-fold increased risk of high LDL-C [34]. However, an epidemiological study conducted in the United States by Kant et al. showed an inverse association between TC, HDL-C, LDL-C and FAFH frequency, but no association with TG, which was different from the results of this study [35]. Compared to Kant’s research, this research subjects mainly focused on middle-aged and older people, who were more exposed to CVD risk factors. Older participants may have increased risk due to long-established FAFH consumption habits.

Tehran Lipid and Glucose Study results showed that increased TG levels and TG accompanied more fast food consumption to HDL-C ratio after three years of follow-up [36]. As the frequency of FAFH increases, there is a corresponding decrease in the frequency of home cooking, and recent studies have shown that teens with low home cooking frequency have lower HDL cholesterol compared with teens with high home cooking frequency (*β* = -6.15, 95% *CI*: -11.2, 1.07). Numerous studies have shown that FAFH provides more sodium, energy, fat and saturated fatty acids, and less personal nutrients and dietary fibers than cooking at home [21]. Additionally, Jessica E Todd found that higher FAFH energy percentages lead to a higher intake of fat and cholesterol. Therefore, it is necessary to increase the intake of fish and plant foods to control dyslipidemia [37, 38].

Although many epidemiological studies pointed out the health risks of skipping breakfast, especially cardiovascular disease risk, few studies have examined the risks associated with breakfast FAFH consumption [39, 40]. This research found that breakfast, lunch and dinner FAFH frequencies were significantly associated with dyslipidemia. Previous study showed that nearly two-thirds of residents purchased FAFH for breakfast and dinner, which were high in refined carbohydrates, and saturated fat, but lacking in protein [41]. An epidemiological study in Brazil showed that participants with higher breakfast quality assessment scores were likelier to maintain a lower level of TC and LDL-C [42]. This unhealthy dietary pattern increases the risk of dyslipidemia [21, 37]. FAFH provides consumers with more food choices, but manufacturers ignore nutrition in pursuit of taste, so FAFH consumers should pay more attention to dietary nutrition. Developing a personalized nutritional treatment plan for patients with dyslipidemia is necessary. Therefore, the Mediterranean and Nordic dietary patterns can effectively interfere with serum lipid levels [43].

The risk of dyslipidemia caused by FAFH was different in different demographic characteristics, living habits and dietary habits in this study. Similar to previous studies, FAFH was mainly distributed among people with a higher average monthly income in this study [44]. In terms of lifestyle habits, this study found that FAFH brought a greater risk of dyslipidemia in smokers and drinkers. Studies showed that smoking stimulates growth hormone secretion, increases TC and TG, increases LDL-C levels, and reduces HDL-C levels [45, 46]. At the same time, numerous studies have shown that drinkers have a higher risk of dyslipidemia, especially for heavy drinkers [47, 48]. This study found that low and moderate physical activity populations bear a greater risk of dyslipidemia due to FAFH. Appropriate and regular physical activity can help reduce the risk of dyslipidemia in people who frequently eat out [49]. Regarding dietary habits, the results showed that people with a high-fat diet had a higher risk of dyslipidemia when they ate out. Since FAFH nutrients tend to be biased toward high-fat components, FAFH may be the mainstay of high-fat-fed populations. Therefore, for people with abnormal lipid metabolism, healthy eating habits have a little but important effect on their blood lipid levels [50].

An interesting point of this study is that BMI fully mediated the relationship between FAFH and dyslipidemia in men. FAFH intake may be associated with high BMI and obesity. The result showed that FAFH frequency was significant association with a higher BMI [51], and a French cohort study also showed that people with high intake of ultra-processed foods got a higher BMI. The energy content and portion size of foods when FAFH may lead to excess energy intake in those who eat out frequently, which may lead to weight gain. Therefore, more research is needed to clarify the mechanism between BMI and dyslipidemia.

Under the cultural background of Chinese rural adults, carrying out nutrition education and popularizing dietary guidelines, training catering workers to improve their food health awareness; providing nutrition information about food on the menu of the restaurant, strengthening the management and supervision of food health and nutrition in restaurants, and improving the quality and nutrition of FAFH may help reduce the behavior of FAFH and health hazards.

## Comparisons with other studies and what does the current work add to the existing knowledge

FAFH may be associated with the development of metabolic diseases. However, the relationship between FAFH and dyslipidemia remains unclear, especially in rural areas. The current study firstly reported a significant association between FAFH frequency and dyslipidemia in rural Chinese adults using a large sample data. The results of this study provide a basis for dietary intervention in clinical dyslipidemia patients.

## Study strength and limitations

The present study has several strengths. This study used a large sample of data from rural China, performed an accurate experimental design and data collection and adjusted for known or potential confounding factors. The results of this study have important implications for the clinical treatment of patients with dyslipidemia and cardiovascular disease. Dietary intervention is one of the important contents of lifestyle intervention for patients with dyslipidemia. By reducing the frequency of eating out to limit high-calorie, high-fat and high-salt intake, the blood lipid level can be effectively controlled without affecting the normal nutritional status of the patient’s body. It helps to improve clinical treatment effects and maintain blood lipid levels at a healthy level.

However, the limitation of this study must be acknowledged. First, this is a cross-sectional study, more research including longitudinal studies as well as multicenter studies should be conducted to confirm the causality between FAFH and dyslipidemia. Future research should focus on the molecular mechanisms of the nutrients in FAFH on dyslipidemia and blood lipid levels, which are important for improving FAFH diet quality. Second, FAFH-related information was obtained through self-reporting, so recall bias was inevitable. Third, although various covariates were adjusted in the analysis, the occurrence of dyslipidemia was affected by many factors, and various unknown potential factors may affect the association results, this study ignored specific nutritional intake information about FAFH in the investigation, such as specific food types and nutritional components.

## Conclusion

This study found that FAFH was significantly associated with higher levels of TC, TG, LDL-C, and lower HDL-C, and was significantly associated with a higher risk of dyslipidemia and its four parameter types in rural China, especially in men. BMI played a fully mediating role between FAFH and dyslipidemia in men. Controlling or reducing the frequency of FAFH can be one of the important measures for food intake intervention in patients with dyslipidemia or cardiovascular disease.

## Electronic supplementary material

Below is the link to the electronic supplementary material.

Supplementary Material 1

## Data Availability

The raw data supporting the conclusions of this article will be made available by the authors, without undue reservation, to any qualified researcher.

## Abbreviations

OR: odds ratios
SD: standard deviation
IQR: interquartile range
CI: confidence interval
FAFH: food-away-from-home
CVD: cardiovascular disease
T2DM: type 2 diabetes mellitus
TC: total cholesterol
TG: triglycerides
HDL − C: high − density lipoprotein cholesterol
LDL − C: low − density lipoprotein cholesterol
BMI: body mass index
WC: waist circumference
RMB: renminbi
