# Supplementary material for: Gender-specific relationship between frequency of food-away-from-home with serum lipid levels and dyslipidemia in chinese rural adults
Source: Lipids Health Dis. 2022 Nov 1;21:112. doi: 10.1186/s12944-022-01719-6 (PMC9623917; doi:10.1186/s12944-022-01719-6)
Supplement: Supplementary file 1 — Supplementary Material 1 [file 12944_2022_1719_MOESM1_ESM.docx]

Supplementary Material

## Supplementary Tables

| **Weekly frequency of**  **FAFH** | High TC | *P* | High TG | *P* | Low HDL-C | *P* | High LDL-C | *P* |
| --- | --- | --- | --- | --- | --- | --- | --- | --- |
|  | *OR* (95*%* *CI*) |  | *OR* (95*%* *CI*) |  | *OR* (95*%* *CI*) |  | *OR* (95*%* *CI*) |  |
| **Total (n = 29,479)** |  |  |  |  |  |  |  |  |
| **0 time (n = 25,833)** | 1 (Ref.) |  | 1 (Ref.) |  | 1 (Ref.) |  | 1 (Ref.) |  |
| **1~3 times (n = 1,371)** | 1.167 (0.934, 1.457) | 0.174 | 1.137 (0.989, 1.037) | 0.070 | 1.263 (1.105, 1.444) | 0.001 | 0.857 (0.661, 1.112) | 0.246 |
| **4~7 times (n = 1,130)** | 1.491 (1.177, 1.888) | 0.001 | 1.130 (0.968, 1.319) | 0.123 | 1.281 (1.1.08, 1.482) | 0.001 | 1.372 (1.072, 1.756) | 0.012 |
| **8~11 times (n = 298)** | 1.928 (1.247, 2.980) | 0.003 | 1.723 (1.321, 2.247) | < 0.001 | 1.513 (1.168, 1.959) | 0.002 | 1.875 (1.215, 2.893) | 0.005 |
| **≥ 12 times (n = 847)** | 1.414 (1.077, 1.857) | 0.013 | 1.132 (0.950, 1.350) | 0.166 | 1.056 (0.889, 1.254) | 0.535 | 1.625 (1.254, 2.105) | < 0.001 |
| **Men (n = 12,002)** |  |  |  |  |  |  |  |  |
| **0 time (n = 9759)** | 1 (Ref.) |  | 1 (Ref.) |  | 1 (Ref.) |  | 1 (Ref.) |  |
| **1~3 times (n = 707)** | 1.227 (0.907, 1.658) | 0.184 | 1.209 (1.006, 1.453) | 0.043 | 1.362 (1.147, 1.616) | < 0.001 | 0.900 (0.643, 1.258) | 0.536 |
| **4~7 times (n = 720)** | 1.268 (0.941, 1.709) | 0.118 | 1.090 (0.904, 1.315) | 0.368 | 1.321 (1.111, 1.570) | 0.002 | 1.217 (0.902, 1.641) | 0.198 |
| **8~11 times (n = 238)** | 1.161 (0.699, 1.929) | 0.563 | 1.438 (1.077, 1.920) | 0.014 | 1.503 (1.136, 1.990) | 0.004 | 1.248 (0.769, 2.026) | 0.369 |
| **≥ 12 times (n = 578)** | 1.339 (0.965, 1.585) | 0.081 | 1.087 (0.884, 1.336) | 0.429 | 1.074 (0.882, 1.307) | 0.479 | 1.426 (1.044, 1.947) | 0.026 |
| **Women (n = 17,477)** |  |  |  |  |  |  |  |  |
| **0 time (n = 16,074)** | 1 (Ref.) |  | 1 (Ref.) |  | 1 (Ref.) |  | 1 (Ref.) |  |
| **1~3 times (n = 664)** | 1.052 (0.751, 1.474) | 0.769 | 1.025 (0.820, 1.283) | 0.826 | 1.162 (0.927, 1.457) | 0.193 | 0.789 (0.517, 1.203) | 0.271 |
| **4~7 times (n = 410)** | 1.416 (0.945, 2.122) | 0.092 | 0.869 (0.641, 1.177) | 0.365 | 1.046 (0.779, 1.406) | 0.763 | 1.254 (0.786, 2.003) | 0.343 |
| **8~11 times (n = 60)** | 2.665 (1.105, 6.431) | 0.029 | 0.741 (0.311, 1.763) | 0.498 | 0.665 (0.264, 1.676) | 0.387 | 2.155 (0.757, 6.135) | 0.150 |
| **≥ 12 times (n = 269)** | 0.843 (0.494, 1.440) | 0.533 | 0.664 (0.452, 0.975) | 0.037 | 0.719 (0.477, 1.084) | 0.115 | 1.188 (0.716, 1.973) | 0.505 |

**Table S1** Multivariate−adjusted *OR* and 95*%* *CI* for abnormal serum lipid levels according to weekly frequency of food-away-from-home

CI confidence interval; OR odds ratio.

Full-adjusted model for age, gender (only for total participants), marital status, education level, average monthly income, smoking status, drinking status, physical activity, vegetable and fruit intake, high fat diet, family history of dyslipidemia.

| **Weekly frequency of**  **FAFH** | **Prevalence, *%* (95*%* *CI*)** | ***OR* (95*%* *CI*)** | | | | ***P* _trend_** |
| --- | --- | --- | --- | --- | --- | --- |
|  |  | **Model 1** | **Model 2** | **Model 3** | ***Per level risk** |  |
| **Total (n = 29,479)** |  |  |  |  | 1.027 (1.005, 1.049) | 0.014 |
| **0 time (n = 27,921)** | 38.21 (37.64, 38.78) | 1 (Ref.) | 1 (Ref.) | 1 (Ref.) |  |  |
| **1~2 times (n = 491)** | 41.75 (37.37, 46.13) | 1.420 (1.179, 1.710) | 1.047 (1.168, 1.695) | 1.398 (1.159, 1.686) |  |  |
| **3~4 times (n = 309)** | 46.28 (40.69, 51.87) | 1.647 (1.309, 2.072) | 1.626 (1.292, 2.047) | 1.611 (1.278, 2.030) |  |  |
| **5~6 times (n = 152)** | 34.87 (27.21, 42.53) | 0.983 (0.701, 1.380) | 0.976 (0.695, 1.371) | 0.997 (0.709, 1.403) |  |  |
| **7 times (n = 606)** | 39.93 (36.02, 43.84) | 1.131 (0.958, 1.336) | 1.127 (0.954, 1.332) | 1.138(0.962, 1.346) |  |  |
| **Men (n = 12,002)** |  |  |  |  | 1.025 (0.998, 1.053) | 0.067 |
| **0 time (n = 11,034)** | 39.22 (38.30, 40.13) | 1 (Ref.) | 1 (Ref.) | 1 (Ref.) |  |  |
| **1~2 times (n = 268)** | 55.97 (49.99, 61.95) | 1.592 (1.241, 2.043) | 1.552 (1.208, 1.993) | 1.559 (1.211, 2.006) |  |  |
| **3~4 times (n = 210)** | 57.14 (50.39, 63.89) | 1.669 (1.262, 2.208) | 1.649 (1.246, 2.184) | 1.640 (1.236, 2.177) |  |  |
| **5~6 times (n = 108)** | 38.89 (29.55, 48.23) | 0.809 (0.547, 1.198) | 0.788 (0.531, 1.168) | 0.809 (0.544, 1.202) |  |  |
| **7 times (n = 382)** | 45.29 (40.27, 50.30) | 1.118 (0.909, 1.376) | 1.107 (0.899, 1.364) | 1.119 (0.907, 1.381) |  |  |
| **Women (n = 17,477)** |  |  |  |  | 0.987 (0.950, 1.026) | 0.499 |
| **0 time (n = 16,887)** | 37.56 (36.83, 38.29) | 1 (Ref.) | 1 (Ref.) | 1 (Ref.) |  |  |
| **1~2 times (n = 223)** | 24.66 (18.96, 30.37) | 0.996 (0.703, 1.327) | 0.995 (0.723, 1.368) | 0.992 (0.720, 1.366) |  |  |
| **3~4 times (n = 99)** | 23.23 (14.77, 31.70) | 0.957 (0.590, 1.551) | 0.971 (0.599, 1.576) | 0.950 (0.584, 1.544) |  |  |
| **5~6 times (n = 44)** | 25.00 (11.68, 38.32) | 0.946 (0.468, 1.912) | 0.986 (0.486, 1.999) | 1.017 (0.501, 2.063) |  |  |
| **7 times (n = 224)** | 30.80 (24.71, 36.90) | 0.958 (0.714, 1.285) | 0.976 (0.727, 1.311) | 0.986 (0.733, 1.327) |  |  |

**Table S2** Multivariate−adjusted *OR* and 95*%* *CI* for dyslipidemia according to weekly frequency of away-from-home breakfasts

CI confidence interval; OR odds ratio.

* Full−adjusted model for age, gender (only for total participants), marital status, education level, average monthly income, smoking status, drinking status, physical activity, vegetable and fruit intake, high fat diet, family history of dyslipidemia;

Model 1: adjusted for age, gender (only for total participants);

Model 2: adjusted for age, gender (only for total participants), marital status, education level, average monthly income, smoking status, drinking status;

Model 3: adjusted for age, gender (only for total participants), marital status, education level, average monthly income, smoking status, drinking status, physical activity, vegetable and fruit intake, high fat diet, family history of dyslipidemia;

Trends of odds ratios were performed using the categories (0 time, 1~2 times, 3~4 times, 5~6 times, 7 times) of weekly frequency of food-away-from-home group as continuous variables in the logistic regression model.

| **Weekly frequency of**  **FAFH** | **Prevalence, *%* (95*% CI*)** | ***OR* (95% *CI*)** | | | | ***P* _trend_** |
| --- | --- | --- | --- | --- | --- | --- |
|  |  | **Model 1** | **Model 2** | **Model 3** | ***Per level risk** |  |
| **Total (n = 29,479)** |  |  |  |  | 1.028 (1.011, 1.045) | 0.001 |
| **0 time (n = 26,819)** | 38.11 (37.53, 38.69) | 1 (Ref.) | 1 (Ref.) | 1 (Ref.) |  |  |
| **1~2 times (n = 861)** | 41.58 (38.28, 44.88) | 1.289 (1.119, 1.484) | 1.278 (1.109, 1.473) | 1.267 (1.099, 1.460) |  |  |
| **3~4 times (n = 448)** | 46.43 (41.79, 51.06) | 1.568 (1.295, 1.899) | 1.544 (1.274, 1.871) | 1.516 (1.249, 1.839) |  |  |
| **5~6 times (n = 278)** | 41.37 (35.54, 47.19) | 1.341 (1.050, 1.713) | 1.332 (1.042, 1.702) | 1.330 (1.039, 1.702) |  |  |
| **7 times (n = 1,073)** | 38.30 (35.39, 41.22) | 1.107 (0.973, 1.259) | 1.101 (0.967, 1.253) | 1.120 (0.984, 1.276) |  |  |
| **Men (n = 12,002)** |  |  |  |  | 1.032 (1.010, 1.053) | 0.004 |
| **0 time (n = 10,271)** | 38.62 (37.68, 39.57) | 1 (Ref.) | 1 (Ref.) | 1 (Ref.) |  |  |
| **1~2 times (n = 514)** | 48.64 (44.30, 52.97) | 1.258 (1.050, 1.508) | 1.241 (1.034, 1.489) | 1.253 (1.043, 1.505) |  |  |
| **3~4 times (n = 327)** | 55.66 (50.24, 61.07) | 1.654(1.321, 2.072) | 1.635 (1.303, 2.051) | 1.602 (1.274, 2.014) |  |  |
| **5~6 times (n = 202)** | 47.52 (40.58, 54.47) | 1.125(0.847, 1.494) | 1.111 (0.835, 1.478) | 1.095 (0.821, 1.461) |  |  |
| **7 times (n = 688)** | 46.08 (42.34, 49.81) | 1.135(0.968, 1.330) | 1.123 (0.957, 1.318) | 1.158 (0.985, 1.361) |  |  |
| **Women (n = 17,477)** |  |  |  |  | 0.958 (0.929, 0.989) | 0.008 |
| **0 time (n = 16,548)** | 37.79 (37.05, 38.53) | 1 (Ref.) | 1 (Ref.) | 1 (Ref.) |  |  |
| **1~2 times (n = 347)** | 31.12 (26.23, 36.02) | 1.115 (0.878, 1.415) | 1.148 (0.903, 1.459) | 1.120 (0.880, 1.426) |  |  |
| **3~4 times (n = 121)** | 21.49 (14.06, 28.91) | 0.730 (0.468, 1.140) | 0.754 (0.482, 1.178) | 0.753 (0.481, 1.179) |  |  |
| **5~6 times (n = 76)** | 25.00 (15.04, 34.96) | 0.974 (0.570, 1.664) | 1.009 (0.590, 1.728) | 1.046 (0.610, 1.795) |  |  |
| **7 times (n = 385)** | 24.42 (20.11, 28.73) | 0.735 (0.578, 0.936) | 0.742 (0.583, 0.945) | 0.750 (0.588, 0.956) |  |  |

**Table S3** Multivariate−adjusted *OR* and 95*%* *CI* for dyslipidemia according to weekly frequency of away-from-home lunches

CI confidence interval; OR odds ratio.

* Full−adjusted model for age, gender (only for total participants), marital status, education level, average monthly income, smoking status, drinking status, physical activity, vegetable and fruit intake, high fat diet, family history of dyslipidemia;

Model 1: adjusted for age, gender (only for total participants);

Model 2: adjusted for age, gender (only for total participants), marital status, education level, average monthly income, smoking status, drinking status;

Model 3: adjusted for age, gender (only for total participants), marital status, education level, average monthly income, smoking status, drinking status, physical activity, vegetable and fruit intake, high fat diet, family history of dyslipidemia;

Trends of odds ratios were performed using the categories (0 time, 1~2 times, 3~4 times, 5~6 times, 7 times) of weekly frequency of food-away-from-home group as continuous variables in the logistic regression model.

| **Weekly frequency of**  **FAFH** | **Prevalence, *%* (95*% CI*)** | ***OR* (95*%* *CI*)** | | | | ***P* _trend_** |
| --- | --- | --- | --- | --- | --- | --- |
|  |  | **Model 1** | **Model 2** | **Model 3** | ***Per level risk** |  |
| **Total (n = 29,479)** |  |  |  |  | **1.048 (1.027, 1.070)** | **< 0.001** |
| **0 time (n = 27,431)** | 38.09 (37.51, 38.66) | 1 (Ref.) | 1 (Ref.) | 1 (Ref.) |  |  |
| **1~2 times (n = 835)** | 39.64 (36.32, 42.97) | 1.311 (1.133, 1.517) | 1.300 (1.123, 1.506) | 1.275 (1.100, 1.478) |  |  |
| **3~4 times (n = 411)** | 48.18 (43.32, 53.03) | 1.825 (1.493, 2.231) | 1.806 (1.477, 2.210) | 1.761 (1.437, 2.158) |  |  |
| **5~6 times (n = 210)** | 50.95 (44.14, 57.77) | 2.092(1.585, 2.762) | 2.078 (1.574, 2.744) | 2.082 (1.574, 2.755) |  |  |
| **7 times (n = 592)** | 38.51 (34.58, 42.44) | 1.138(0.959, 1.349) | 1.133 (0.955, 1.343) | 1.121 (0.944, 1.331) |  |  |
| **Men (n = 12,002)** |  |  |  |  | **1.032 (1.006, 1.060)** | **0.015** |
| **0 time (n = 10,647)** | 38.70 (37.77, 39.62) | 1 (Ref.) | 1 (Ref.) | 1 (Ref.) |  |  |
| **1~2 times (n = 479)** | 51.98 (47.49, 56.47) | 1.379 (1.141, 1.667) | 1.356 (1.120, 1.641) | 1.328 (1.096, 1.610) |  |  |
| **3~4 times (n = 323)** | 56.35 (50.91, 61.78) | 1.622 (1.290, 2.039) | 1.592 (1.263, 2.005) | 1.536 (1.217, 1.939) |  |  |
| **5~6 times (n = 164)** | 52.44 (44.72, 60.16) | 1.383 (1.010, 1.895) | 1.366 (0.996, 1.874) | 1.346 (0.978, 1.853) |  |  |
| **7 times (n = 389)** | 44.99 (40.02, 49.95) | 1.091 (0.887, 1.343) | 1.083 (0.879, 1.334) | 1.063(0.862, 1.312) |  |  |
| **Women (n = 17,477)** |  |  |  |  | 0.971 (0.932, 1.011) | 0.153 |
| **0 time (n = 16,784)** | 37.70 (36.97, 38.44) | 1 (Ref.) | 1 (Ref.) | 1 (Ref.) |  |  |
| **1~2 times (n = 356)** | 23.03 (18.64, 27.43) | 0.872 (0.673, 1.130) | 0.896 (0.690, 1.163) | 0.882 (0.678, 1.147) |  |  |
| **3~4 times (n = 88)** | 18.18 (9.96, 26.40) | 0.727 (0.418, 1.267) | 0.762 (0.437, 1.329) | 0.765 (0.437, 1.336) |  |  |
| **5~6 times (n = 46)** | 45.65 (30.70, 60.61) | 2.345 (1.277, 4.305) | 2.393 (1.301, 4.401) | 2.450 (1.328, 4.522) |  |  |
| **7 times (n = 203)** | 26.11 (20.01, 32.20) | 0.765 (0.554, 1.057) | 0.771 (0.558, 1.065) | 0.762 (0.550, 1.054) |  |  |

**Table S4** Multivariate−adjusted *OR* and 95*% CI* for dyslipidemia according to weekly frequency of away-from-home dinner

*CI* confidence interval; *OR* odds ratio.

* Full−adjusted model for age, gender (only for total participants), marital status, education level, average monthly income, smoking status, drinking status, physical activity, vegetable and fruit intake, high fat diet, family history of dyslipidemia;

Model 1: adjusted for age, gender (only for total participants);

Model 2: adjusted for age, gender (only for total participants), marital status, education level, average monthly income, smoking status, drinking status;

Model 3: adjusted for age, gender (only for total participants), marital status, education level, average monthly income, smoking status, drinking status, physical activity, vegetable and fruit intake, high fat diet, family history of dyslipidemia;

Trends of odds ratios were performed using the categories (0 time, 1~2 times, 3~4 times, 5~6 times,7 times) of weekly frequency of food-away-from-home group as continuous variables in the logistic regression model.

| **Weekly frequency of**  **FAFH** | **Model 1** | | | **Model 2** | | | **Model 3** | | | **Per level risk**^a^ | | |
| --- | --- | --- | --- | --- | --- | --- | --- | --- | --- | --- | --- | --- |
|  | *β* | 95*% CI* | *P* | *β* | 95*% CI* | *P* | *β* | 95*% CI* | *P* | *β* | 95*% CI* | *P* |
| **Men (n = 12,002)** |  |  |  |  |  |  |  |  |  | 0.186 | (0.124, 0.249) | < 0.001 |
| **0 time (n = 9759)** | 0 (Ref.) |  |  | 0 (Ref.) |  |  | 0 (Ref.) |  |  |  |  |  |
| **1~3 times (n = 707)** | 0.938 | (0.344, 0.926) | < 0.001 | 0.821 | (0.560, 1.081) | < 0.001 | 0.804 | (0.544, 1.063) | < 0.001 |  |  |  |
| **4~7 times (n = 720)** | 0.748 | (0.496, 1.383) | < 0.001 | 0.621 | (0.359, 0.883) | < 0.001 | 0.605 | (0.344, 0.866) | < 0.001 |  |  |  |
| **8~11 times (n = 238)** | 0.939 | (0.483, 1.013) | <0.001 | 0.765 | (0.326, 1.203) | 0.001 | 0.720 | (0.282, 1.158) | 0.001 |  |  |  |
| **≥ 12 times (n = 578)** | 0.635 | (0.675, 1.202) | < 0.001 | 0.529 | (0.241, 0.817) | < 0.001 | 0.517 | (0.229, 0.806) | < 0.001 |  |  |  |
| **Women (n = 17,477)** |  |  |  |  |  |  |  |  |  | 0.062 | (−0.027, 0.151) | 0.174 |
| **0 time (n = 16,074)** | 0 (Ref.) |  |  | 0 (Ref.) |  |  | 0 (Ref.) |  |  |  |  |  |
| **1~3 times (n = 664)** | 0.040 | (-0.246, 0.326) | 0.784 | 0.128 | (-0.159, 0.415) | 0.382 | 0.098 | (-0.189, 0.384) | 0.504 |  |  |  |
| **4~7 times (n = 410)** | 0.326 | (-0.033, 0.685) | 0.075 | 0.409 | (0.050, 0.768) | 0.026 | 0.376 | (0.017, 0.734) | 0.040 |  |  |  |
| **8~11 times (n = 60)** | -0.262 | (-1.177, 0.653) | 0.574 | -0.144 | (-1.058, 0.770) | 0.758 | -0.187 | (-1.100, 0.725) | 0.688 |  |  |  |
| **≥ 12 times (n = 269)** | 0.020 | (-0.415, 0.456) | 0.927 | 0.108 | (-0.328, 0.543) | 0.628 | 0.119 | (-0.316, 0.554) | 0.592 |  |  |  |

**Table S5** Multivariate−adjusted *β*−coefficients and 95*%* *CI* for BMI according to the weekly frequency of food-away-from-home

*CI* confidence interval;

^a^ Full−adjusted model for age, marital status, education level, average monthly income, smoking status, drinking status, physical activity, vegetable and fruit intake, high fat diet, family history of dyslipidemia;

Model 1: adjusted for age;

Model 2: adjusted for age, marital status, education level, average monthly income, smoking status, drinking status;

Model 3: adjusted for age, marital status, education level, average monthly income, smoking status, drinking status, physical activity, vegetable and fruit intake, high fat diet, family history of dyslipidemia.

| **Population** | ***OR* (95*%* *CI*)** | ***P*** |
| --- | --- | --- |
|  |  |  |
| **Gender** |  |  |
| **Men** | 1.014 (1.004, 1.024) | 0.004 |
| **Women** | 0.990 (0.975, 1.004) | 0.159 |
| **Age** |  |  |
| **≤60years** | 1.002 (0.993, 1.010) | 0.675 |
| **>60years** | 1.025 (1.006, 1.045) | 0.011 |
| **Averaged monthly income** |  |  |
| **<500RMB** | 1.029 (1.013, 1.046) | 0.001 |
| **500–999 RMB** | 1.010 (0.996, 1.025) | 0.170 |
| **≥1000RMB** | 1.014 (1.003, 1.025) | 0.015 |
| **Smoking status** |  |  |
| **Never** | 1.005 (0.994, 1.016) | 0.370 |
| **Ever** | 1.000 (0.975, 1.025) | 0.993 |
| **Current** | 1.019 (1.006, 1.032) | 0.005 |
| **Drinking status** |  |  |
| **Never** | 1.004 (0.994, 1.014) | 0.452 |
| **Ever** | 1.068 (1.029, 1.109) | 0.001 |
| **Current** | 1.014 (1.001, 1.028) | 0.034 |
| **Physical activity** |  |  |
| **Low** | 1.022 (1.008, 1.036) | 0.003 |
| **Moderate** | 1.017 (1.005, 1.030) | 0.008 |
| **High** | 1.013 (0.999, 1.027) | 0.068 |
| **Vegetable and fruit intake** |  |  |
| **<500g/d** | 1.009 (0.998, 1.020) | 0.094 |
| **≥500g/d** | 1.027 (1.015, 1.038) | < 0.001 |
| **High fat diet** |  |  |
| **Yes** | 1.027 (1.014, 1.040) | < 0.001 |
| **No** | 1.009 (0.999, 1.019) | 0.084 |
| **Family history of dyslipidemia** |  |  |
| **Yes** | 1.009 (0.974, 1.045) | 0.630 |
| **No** | 1.018 (1.010, 1.026) | < 0.001 |

**Table S6** Stratified analysis of the association between food-away-from-home frequency and dyslipidemia

*CI* confidence interval; *OR* odds ratio.

Adjusted for age, gender, marital status, education level, average monthly income, smoking status, drinking status, physical activity, vegetable and fruit intake, high fat diet, family history of dyslipidemia.

**Table S7** Characteristics of participants according by gender

| **Characteristics** | **Men (*n* = 12,002)** | **Women (*n* = 17,477)** | ***P*** |
| --- | --- | --- | --- |
| **Age (mean ± SD)** | 56.5 ± 12.4 | 54.7 ± 12.3 | **<** 0.001 |
| **Marital status, *n* (*%*)** |  |  | 0.751 |
| Married/cohabitation | 1,0824 (90.2) | 1,5742 (90.1) |  |
| Unmarried/divorced/widowed | 1178 (9.8) | 1735 (9.9) |  |
| **Education level, *n* (*%*)** |  |  | **<** 0.001 |
| Elementary school or below | 9430 (78.6) | 15093 (86.4) |  |
| Junior high school | 2122 (17.7) | 1828 (10.5) |  |
| High school or above | 450 (3.7) | 556 (3.2) |  |
| **Averaged monthly income, *n* (*%*)** |  |  | 0.016 |
| <500RMB | 4393 (36.6) | 6240 (35.7) |  |
| 500–999 RMB | 3670 (30.6) | 5620 (32.2) |  |
| ≥1000RMB | 3939 (32.8) | 5617 (32.1) |  |
| **Smoking, *n* (*%*)** |  |  | **<** 0.001 |
| Never | 3821 (31.8) | 1,7409 (99.6) |  |
| Ever | 2296 (19.1) | 18 (0.1) |  |
| Current | 5885 (49.0) | 50 (0.3) |  |
| **Drinking, *n* (*%*)** |  |  | **<** 0.001 |
| Never | 5806 (48.4) | 1,7099 (97.8) |  |
| Ever | 1410 (11.7) | 39 (0.2) |  |
| Current | 4786 (39.9) | 339 (1.9) |  |
| **Physical activity, *n* (*%*)** |  |  | **<** 0.001 |
| Low | 4141 (34.5) | 5230 (29.9) |  |
| Moderate | 3378 (28.1) | 7466 (42.7) |  |
| High | 4483 (37.4) | 4781 (27.4) |  |
| **Vegetable and fruit intake, *n* (*%*)** |  |  | 0.808 |
| <500g/d | 6250 (52.1) | 9127 (52.2%) |  |
| ≥500g/d | 5751 (47.9) | 8350 (47.8) |  |
| **High fat diet, *n* (*%*)** | 2839 (23.7) | 2508 (14.4) | **<** 0.001 |
| **Family history of dyslipidemia, *n* (*%*)** | 346 (2.9) | 730 (4.2) | **<** 0.001 |
| **BMI (mean ± SD)** | 24.5 ± 3.5 | 24.9 ± 3.6 | **<** 0.001 |
| **WC (mean ± SD)** | 85.3 ± 10.6 | 82.5 ± 10.2 | **<** 0.001 |
| **Frequency of FAFH (times/week)** | 1.5 ± 4.0 | 0.5 ± 2.4 | **<** 0.001 |

Continuous variables are presented as mean ± SD; categorical variables are shown as percentages (*%*)

*BMI* body mass index; *WC* waist circumference; *RMB* renminbi; *FAFH* food-away-from-home, *SD* standard deviation

**Table S8** Multivariate-adjusted *OR* and 95*%* CI for dyslipidemia according to food-away-from-home

| **Variables** | ***OR* (95*% CI*)** | | |
| --- | --- | --- | --- |
|  | **Model 1** | **Model 2** | **Model 3** |
| **Total** |  |  |  |
| **FAFH-NO** | 1 (Ref.) | 1 (Ref.) | 1 (Ref.) |
| **FAFH-YES** | 1.275 (1.181, 1.377) | 1.264 (1.170, 1.367) | 1.255 (1.160, 1.358) |
| **Men** |  |  |  |
| **FAFH-NO** | 1 (Ref.) | 1 (Ref.) | 1 (Ref.) |
| **FAFH-YES** | 1.276 (1.155, 1.410) | 1.262 (1.040, 1.397) | 1.260 (1.137, 1.397) |
| **Women** |  |  |  |
| **FAFH-NO** | 1 (Ref.) | 1 (Ref.) | 1 (Ref.) |
| **FAFH-YES** | 0.958 (0.841, 1.092) | 0.979 (0.858, 1.116) | 0.969 (0.849, 1.106) |

*OR*, odd ratio; *CI*, confidence interval;

Model 1: adjusted for age, gender (only for total participants);

Model 2: adjusted for age, gender (only for total participants), marital status, education level, average monthly income, smoking status, drinking status;

Model 3: adjusted for age, gender (only for total participants), marital status, education level, average monthly income, smoking status, drinking status, physical activity, vegetable and fruit intake, high fat diet, family history of dyslipidemia.

**Table S9** Interaction analysis between food-away-from-home frequency and gender on the serum lipids levels^a^

| **Serum lipid levels** | **FAFH direct effect**^b^ | ***P*** | **FAFH × gender interaction effect**^c^ | ***P*** _FAFH-gender_ |
| --- | --- | --- | --- | --- |
| **TC, mmol/L** | 0.200 (0.200, 0.300) | < 0.001 | 0.401 (0.200, 0.501) | < 0.001 |
| **TG, mmol/L** | 0.200 (0.000, 0.400) | 0.120 | 2.224 (1.816, 2.634) | < 0.001 |
| **H-LDL, mmol/L** | -0.100 (-0.200, 0.000) | < 0.001 | -0.803 (-0.995, -0.598) | < 0.001 |
| **L-LDL,** **mmol/L** | 0.300 (0.100, 0.401) | < 0.001 | 0.501 (0.300, 0.702) | < 0.001 |

^a^ Direct and interactive effects were analyzed by generalized linear regression models and expressed as %changes (95%*CI*).

^b^ Adjusted for age, gender, marital status, education level, average monthly income, smoking status, drinking status, physical activity, vegetable and fruit intake, high fat diet, family history of dyslipidemia.

^c^ In addition to all variables adjusted for direct effects, FAFH frequency was included.

**Table S10** Multivariate adjusted *OR*s for dyslipidemia and its four parameter types according to food-away-from-home frequency after adjusting for region

| **Weekly frequency of**  **FAFH** | **Dyslipidemia** | **High TC** | **High TG** | **High LDL-C** | **Low HDL-C** |
| --- | --- | --- | --- | --- | --- |
|  | ***OR* (95*%* *CI*)** | ***OR* (95*%* *CI*)** | ***OR* (95*%* *CI*)** | ***OR* (95*%* *CI*)** | ***OR* (95*%* *CI*)** |
| Total (*n* = 29,479) |  |  |  |  |  |
| 0 time (*n* = 25,833) | 1.00 (Ref.) | 1.00 (Ref.) | 1.00 (Ref.) | 1.00 (Ref.) | 1.00 (Ref.) |
| 1~3 times (*n* = 1,371) | 1.050 (0.934, 1.181) | 0.996 (0.771, 1.210) | 1.135 (0.987, 1.305) | 1.151 (1.006, 1.317) | 0.839 (0.646, 1.090) |
| 4~7 times (*n* = 1,130) | 1.158 (1.018, 1.317) | 1.114 (0.878, 1.415) | 1.135 (0.970, 1.327) | 1.111 (0.959, 1.287) | 1.296 (1.009, 1.663) |
| 8~11 times (*n* = 298) | 1.799 (1.417, 2.285) | 1.427 (0.919, 2.215) | 1.731 (1.326, 2.259) | 1.330 (1.026, 1.725) | 1.740 (1.125, 2.693) |
| ≥ 12 times (*n* = 847) | 1.178 (1.018, 1.363) | 1.264 (0.959, 1.666) | 1.133 (0.950, 1.351） | 0.977 (0.821, 1.162) | 1.601 (1.233, 2.078) |

Abbreviations: TC, total cholesterol; TG, triglycerides; LDL-C, low-density lipoprotein cholesterol; HDL-C, high-density lipoprotein cholesterol; *OR*, odd ratio; *CI,* confidence interval; Ref*,* reference;

Adjusted for age, gender, marital status, education level, average monthly income, smoking status, drinking status, physical activity, vegetable and fruit intake, high fat diet, family history of dyslipidemia, and region.

**Table S11** Associations between weekly frequency of food-away-from-home and blood lipid levels after adjusting for region

| **Weekly frequency of**  **FAFH** | **TC** | **TG** | **HDL-C** | **LDL-C** |
| --- | --- | --- | --- | --- |
|  | *%* Changes (9*5% CI*) | *%* Changes (9*5% CI*) | *%* Changes (9*5% CI*) | *%* Changes (9*5% CI*) |
| Total (*n* = 29,479) |  |  |  |  |
| 0 time (*n* = 25,833) | Ref. | Ref. | Ref. | Ref. |
| 1~3 times (*n* = 1,371) | -0.209 (-1.261, 0.842) | 1.196 (-0.961, 4.884) | -0.930 (-2.291, 0.431) | -0.319 (-1.939, 1.301) |
| 4~7 times (*n* = 1,130) | 0.953 (-0.214, 2.120) | 4.674 (1.430, 7.918) | -0.753 (-2.264, 0.757) | 2.726 (0.928, 4.524) |
| 8~11 times (*n* = 298) | 3.818 (1.632, 6.005) | 16.312 (10.312, 7.918) | -2.229 (-5.060, 0.630) | 3.448 (0.078, 6.818) |
| ≥ 12 times (*n* = 847) | 1.270 (-0.049, 2.590) | 1.138 (-2.531, 4.807) | 0.362 (-1.346, 2.071) | 3.475 (1.441, 5.508) |

Abbreviations: TC, total cholesterol; TG, triglycerides; LDL-C, low-density lipoprotein cholesterol; HDL-C, high-density lipoprotein cholesterol; *CI,* confidence interval; Ref, reference;

Adjusted for age, gender, marital status, education level, average monthly income, smoking status, drinking status, physical activity, vegetable and fruit intake, high fat diet, family history of dyslipidemia, and region.

**
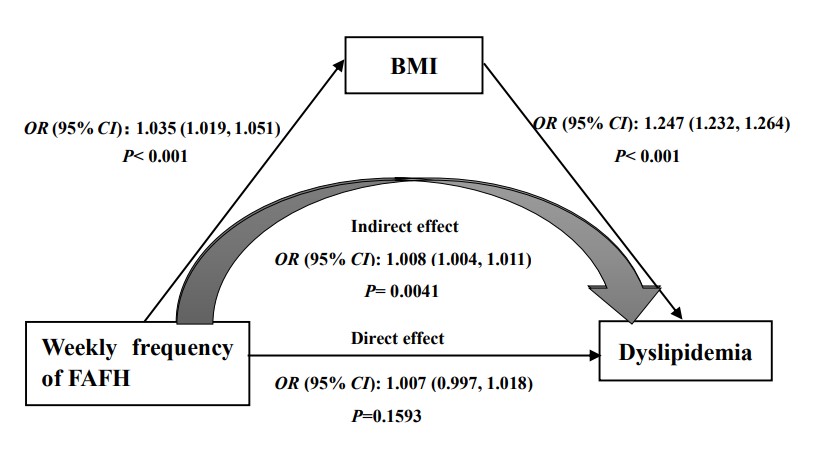
**

**Supplementary Fig. 1** Mediation analysis of the relationship between food-away-from-home (FAFH) frequency and dyslipidemia by BMI in men. BMI body mass index; *CI* confidence interval; *OR* odds ratio. Adjusted for age, marital status, education level, average monthly income, smoking status, drinking status, physical activity, vegetable and fruit intake, high fat diet, family history of dyslipidemia.
